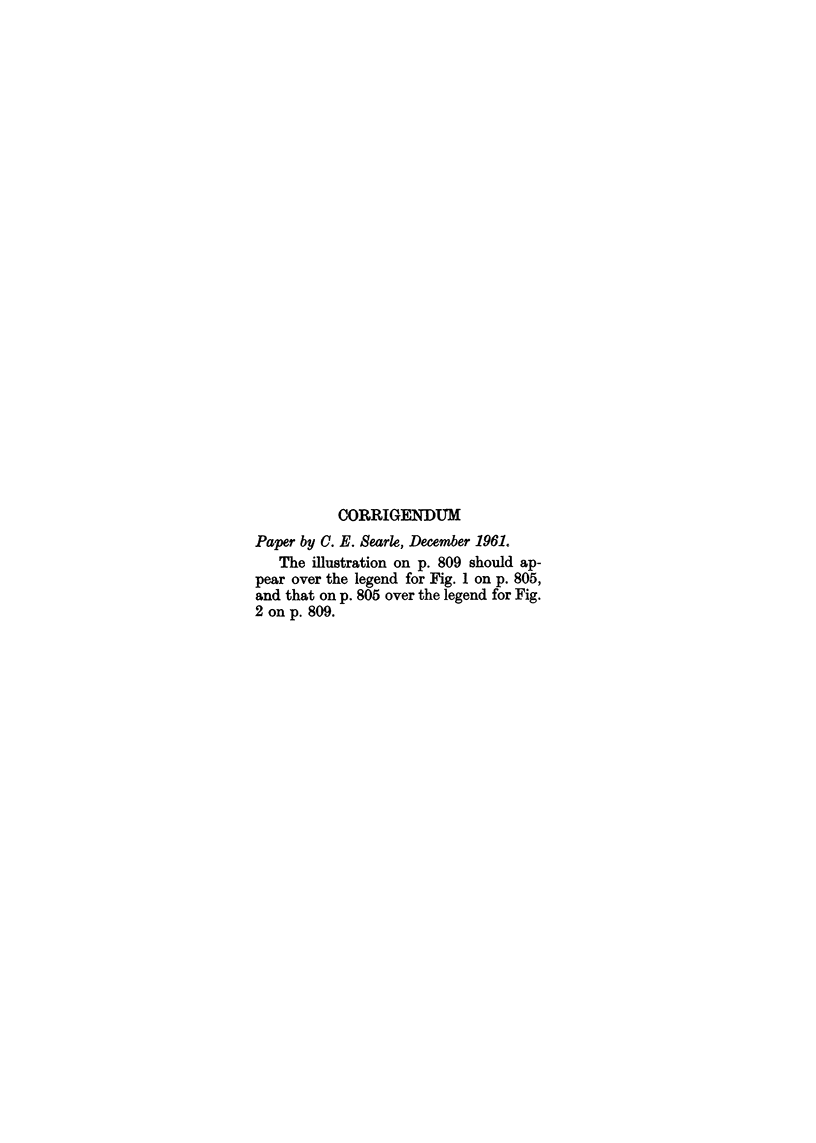# Corrigendum

**Published:** 1962-03

**Authors:** 


### CORRIGENDUM

*Paper by C. E. Searle, December 1961.*

The illustration on p. 809 should appear over the legend for Fig. 1 on p. 805, and that on p. 805 over the legend for Fig. 2 on p. 809.